# Effects of cilostazol against the progression of carotid IMT in symptomatic ischemic stroke patients

**DOI:** 10.1007/s00415-012-6599-y

**Published:** 2012-07-21

**Authors:** Sung Hyuk Heo, Ji Sung Lee, Beom Joon Kim, Kyoung Jin Hwang, Jun-Hyun Kim, Dae-Il Chang

**Affiliations:** 1 Department of Neurology, Kyung Hee University Hospital, #1, Hoegi-dong, Dongdaemun-gu, Seoul, 130-702 Korea; 2 Department of Biostatistics, Korea University, Seoul, Korea; 3 Department of Neurology, Seoul National University Bundang Hospital, Seongnam-si, Gyeonggi-do Korea

**Keywords:** Stroke, Carotid intima-media thickness, Atherosclerosis, Cilostazol

## Abstract

Carotid intima-media thickness (IMT) is a surrogate marker for evaluating atherosclerotic vascular diseases. The phosphodiesterase inhibitor cilostazol attenuates the increase in carotid IMT in diabetes patients. We studied whether cilostazol can reduce the progression of carotid IMT in symptomatic ischemic stroke patients. From our prospective registry of acute ischemic stroke patients who were admitted during a 4.5-year period, follow-up carotid ultrasound was performed in a random sample of survivors. Patients were divided into two groups: the cilostazol group, who continued cilostazol treatment during the follow-up period; and the control group, who were prescribed antiplatelets other than cilostazol. Analysis of covariance and propensity score-matched analysis were used to evaluate the difference between groups. Among a total of 1,049 cases in our registry, 208 patients were utilized to construct two comparable sets by propensity score analysis, including 101 who received cilostazol and 107 who took antiplatelet medication without cilostazol. Both maximum and mean carotid IMT values were significantly reduced in the cilostazol group but increased in the control group (maximum left –0.048 ± 0.186 vs. 0.022 ± 0.163 mm, *p* = 0.001; maximum right –0.037 ± 0.173 vs. 0.050 ± 0.200 mm, *p* = 0.001; mean left –0.052 ± 0.102 vs. 0.023 ± 0.112 mm, *p* < 0.001; and mean right –0.038 ± 0.106 vs. 0.042 ± 0.139 mm, *p* < 0.001). After matching by propensity score, the improvements in both maximum and mean carotid IMT values in the cilostazol group remained significant. This study shows that cilostazol causes a significant regression in carotid IMT in symptomatic stroke patients.

**Electronic supplementary material:**

The online version of this article (doi:10.1007/s00415-012-6599-y) contains supplementary material, which is available to authorized users.

## Introduction

Carotid intima-media thickness (IMT) reflects a preclinical state of atherosclerosis and can be used as a risk marker for myocardial infarction and stroke [6]. Vascular outcomes can be predicted using carotid IMT, and increased carotid IMT values are associated with a higher risk of long-term stroke recurrence [21, 33]. Carotid IMT has been used as a surrogate marker for evaluating therapeutic interventions in atherosclerotic disease [3, 26].

Cilostazol, a phosphodiesterase 3 inhibitor, has been shown to be an alternative to aspirin for secondary prevention in patients with non-cardioembolic ischemic stroke [14, 15]. In addition, cilostazol is suggested to attenuate the increase in carotid IMT in diabetes and coronary patients [1, 16, 24, 28], and it has been reported to prevent the progression of symptomatic intracranial arterial stenosis [18].

In the Cilostazol Against the Progression of carotid Intima-media Thickness in symptomAtic ischemic stroke patients (CAPITA) study, our aim is to assess the effects of cilostazol on the prevention of progression of carotid artery IMT after ischemic stroke.

## Methods

### Subjects and study design

The study design is a hybrid retrospective and prospective study. We retrospectively enrolled symptomatic ischemic stroke or transient ischemic attack (TIA) patients and prospectively obtained follow-up carotid ultrasounds between 12 and 60 months. From January 2005 to June 2009, we identified 1,049 consecutive patients who visited our hospital (<7 days after onset) and who underwent carotid ultrasound.

We excluded subjects who: (1) were younger than 40 or older than 80 years of age; (2) had taken warfarin at discharge; (3) had total internal carotid artery (ICA) occlusion; if the stroke mechanism was (4) cardioembolism or (5) other determined etiologies according to TOAST classification; and (6) if the functional ranking at discharge was greater than 4 on a modified Rankin scale (mRS). After allocation into cilostazol and control groups, additional patients were excluded if they (7) underwent carotid interventions; (8) were lost during follow-up; (9) had not taken medication for more than 80 % of the follow-up period; (10) had taken prohibited concomitant medications such as warfarin, sarpogrelate, beraprost, or mesoglycan; or (11) changed their antiplatelet regimen within the follow-up period. Additionally, if they refused informed consent or if their mRS was four or greater, they were also excluded. In both groups, we randomly contacted the patients by telephone until we had examined more than 100 patients.

All patients maintained antiplatelet therapy after discharge. In the cilostazol group, we selected patients who had taken between 100 and 200 mg/day cilostazol, and in the control group, antiplatelets were restricted to aspirin (100–300 mg/day), clopidogrel (75 mg/day), or triflusal (300–900 mg/day), all of which are commonly used in Korea. We did not exclude patients who received additional antiplatelet therapy in the cilostazol group (Supplementary Table 1). The primary endpoint of this study was changes in the mean and maximum common carotid artery IMT (CCA-IMT) between baseline and follow-up. The secondary endpoint was the occurrence of vascular events including ischemic stroke, TIA, acute coronary syndrome, or hemorrhagic stroke and hemorrhage requiring hospital admission and/or transfusion. Laboratory data such as lipid profile, hsCRP, and glycated hemoglobin were also collected.

All subjects provided informed consent, and the study was approved by the institutional review board at our hospital (KMC IRB 0849-02).

### Measurement of IMT

Ultrasonography was performed on the left and right carotid arteries using the GE Logiq 7 Ultrasound System (GE Medical Systems, Milwaukee, WI, USA) throughout the entire follow-up period. Standardized longitudinal B-mode images with a 12-MHz linear transducer were obtained from the near and far walls of the common carotid arteries. We used the method recommended by Mannheim carotid IMT consensus [32]. However, because the majority of the currently available evidence addressing the assumption of carotid IMT as an indicator of generalized atherosclerosis comes from studies where regular thickening and plaques were included in the IMT measurement, we included IMT (plaques) data if the thickness was greater than 0.5 mm [23].

Carotid ultrasonography was performed by an expert sonographer who was unaware of the clinical information of the subjects. Baseline carotid ultrasound measurements were performed within a few days after stroke onset, and follow-up measurements were prospectively performed between October 2008 and September 2010 (12–60 months after baseline exam).

To avoid inter-reader variability, all IMT measurements were read by two observers (Hwang KJ and Kim JH) blinded to the clinical information, using the semi-automated digital innovative measurement software, Intimascope (Media Cross Co. Ltd., Tokyo, Japan) [34]. The IMT was measured as the distance between two parallel lines corresponding to the lumen-intima and media-adventitia interface on the posterior walls of the common carotid arteries. The maximum IMT values of both common carotid arteries (maximum CCA-IMT) and the mean CCA-IMT were calculated by Intimascope (Supplementary Fig 1). We adopted the mean values of maximum CCA-IMT and mean CCA-IMT read by two independent observers. The interclass correlation coefficients of both maximum CCA-IMT and mean CCA-IMT were 0.812–0.912 (Cronbach’s alpha 0.912–0.961).

### Statistical analysis

We assumed that the differences in CCA-IMT between groups would be 0.08 mm over the 2 years [16, 24]. Therefore, registration of at least 98 patients was required to obtain 80 % power to detect a difference of 0.08 mm in CCA-IMT, assuming a standard deviation of 0.20, no dropout, and a 0.05 level of significance. In this study, the target number of enrolled patients was set at 100 for each group.

The baseline demographic, clinical, and laboratory variables were compared using Student’s *t* test or Mann–Whitney tests for continuous variables and using Pearson’s Chi-square test or Fisher’s exact tests for categorical ones. Analysis of covariance (ANCOVA) was used to compare the means of primary endpoint between the two groups, with adjustment for potential clinical variables. The following potential clinical variables were selected: age, gender, body mass index, stroke subtype, location, previous stroke history, hypertension, diabetes, hyperlipidemia, current smoking, administration of statin, glycated hemoglobin, total cholesterol, high-density lipoprotein (HDL) cholesterol, triglyceride, low-density lipoprotein (LDL) cholesterol, and the interval between baseline and follow-up carotid ultrasound exam.

Because of the possibility of residual confounding even with multivariable analyses, secondary analyses were performed using a propensity-matched sample. For the propensity score analysis, a multivariable logistic regression model that predicted the cilostazol group was generated. The potential clinical variables were included in the model. The predicted probability of cilostazol group (i.e., propensity score) was then calculated for each patient. A greedy matching algorithm was used to match the cilostazol group’s patients with the control group’s patients within a caliper of 0.6 SD of the logit of the propensity score, with a matching ratio of 1:1 [4, 27]. To determine whether the propensity-score approach achieved balance in potential confounders, we assessed absolute standardized differences for each confounder. Thus, evidence of imbalance in potential confounders was identified by examining the reduction in absolute standardized differences. The adequate balance was defined as absolute standardized difference less than 0.1. In the final propensity-score-matched sample, we compared the primary endpoint between cilostazol and control groups using paired *t* tests.

All statistical analyses were conducted using SPSS for Windows version 18.0 (SPSS Inc., Chicago, IL, USA) and SAS version 9.2 (SAS Institute, Cary, NC, USA), and the level of significance was accepted at *p* < 0.05. All hypothesis tests were two-sided.

## Results

**Fig. 1:**
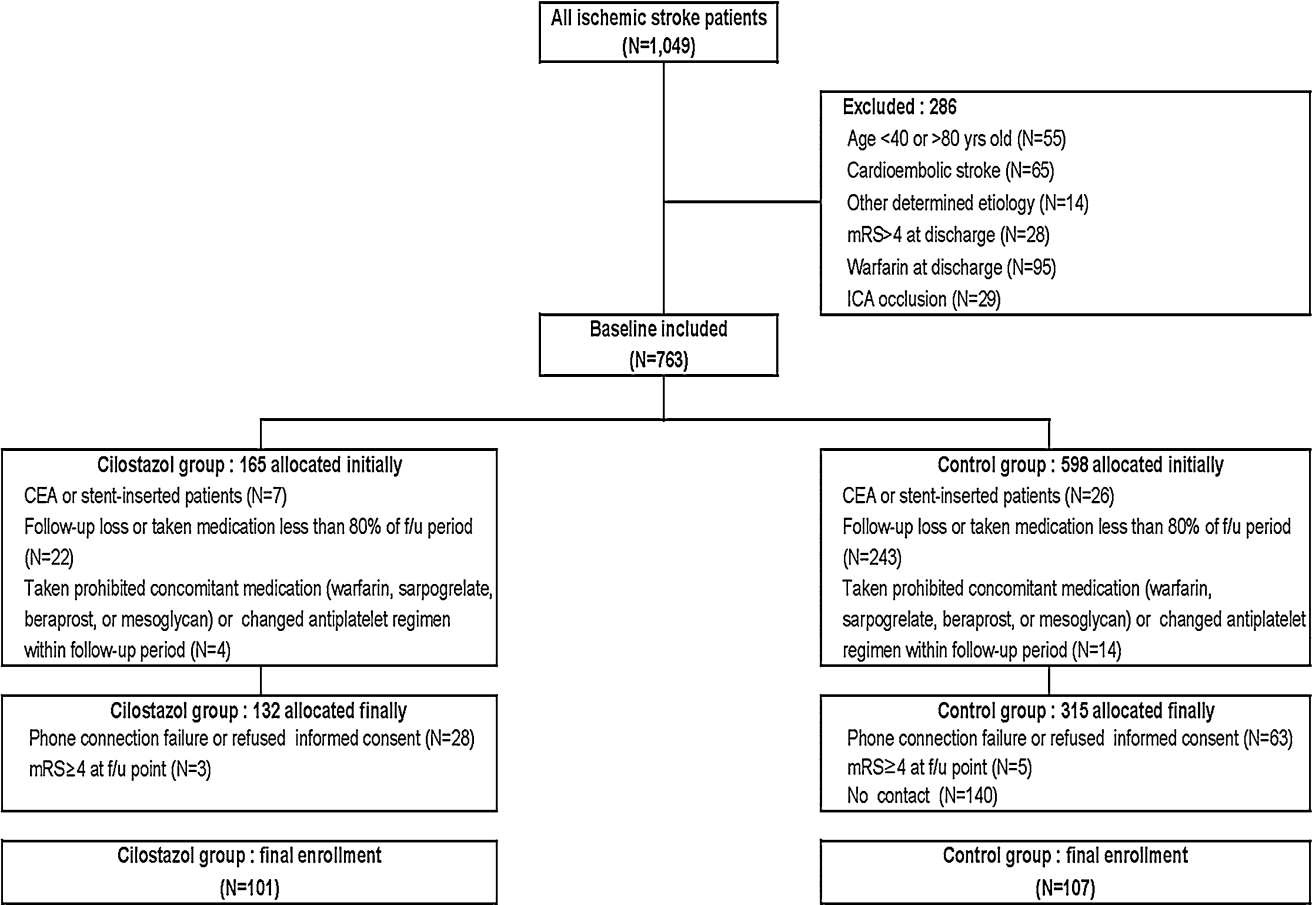
Disposition of patients and reasons for patient exclusion. *mRS* modified Rankin scale, *ICA* internal carotid artery, *CEA* carotid endarterectomy

A total of 447 patients were assigned to the cilostazol and control group (132 and 315 patients, respectively). After the exclusion of 31 and 208 patients in the cilostazol and control groups, respectively, the remaining 101 and 107 patients for each group were included in the final analysis (Fig. 1).

The subjects ranged in age from 40 to 78 years (mean 62.5 ± 8.8 years). The mean interval between baseline and follow-up exam carotid ultrasound was 2.1 years. There were no significant differences in clinical or laboratory findings between the cilostazol and control groups, except the antiplatelet regimen (Table 1).

**Table 1:** Baseline demographics and clinical characteristics of all patients

	Cilostazol (*n* = 101)		Control (*n* = 107)		*p* value^*^
Gender (male), *n* (%)	67	(66.3)	71	(66.4)	0.998
Age (years)	62.76	±9.04	62.26	±8.58	0.682^†^
Body mass index, kg/m^2^	24.47	±2.95	23.92	±2.65	0.161^†^
Stroke subtype, *n* (%)					0.238
LAA	43	(42.6)	37	(34.6)	
SVO	28	(27.7)	40	(37.4)	
Undetermined	17	(16.8)	22	(20.6)	
TIA	13	(12.9)	8	(7.5)	
Location (%)					0.170^‡^
Anterior circulation	67	(66.3)	59	(55.1)	
Posterior circulation	33	(32.7)	47	(43.9)	
Both	1	(1.1)	1	(1.0)	
Previous stroke, *n* (%)	25	(24.8)	19	(17.8)	0.217
Hypertension, *n* (%)	77	(76.2)	76	(71.0)	0.395
Diabetes, *n* (%)	31	(30.7)	30	(28.0)	0.674
Hyperlipidemia, *n* (%)	39	(38.6)	46	(43.0)	0.521
Current smoking, *n* (%)	27	(26.7)	40	(37.7)	0.134
Cilostazol, *n* (%)	101	(100.0)	0	(0.0)	<0.001
100 mg/day	29	(28.7)			
200 mg/day	72	(71.3)			
Statin, *n* (%)	59	(58.4)	59	(55.1)	0.634
Admission NIHSS	3.00	(1–4)	3.00	(1–4)	0.552^§^
Discharge mRS					0.725
0	20	(19.8)	19	(17.8)	
1	44	(43.6)	42	(39.3)	
2	27	(26.7)	30	(28.0)	
3	6	(5.9)	12	(11.2)	
4	4	(4.0)	4	(3.7)	
Hemoglobin A1c, (%)	6.05	±1.23	6.22	±1.37	0.352^†^
Total cholesterol (mg/dl)	188.20	±41.34	190.42	±37.59	0.685^†^
HDL cholesterol (mg/dl)	41.46	±11.00	40.51	±11.02	0.538^†^
Triglyceride (mg/dl)	141.91	±70.43	149.37	±106.87	0.555^†^
LDL cholesterol (mg/dl)	117.41	±35.68	118.07	±33.82	0.890^†^
Interval from baseline to follow-up carotid ultrasound (months)	23.88	±11.95	26.19	±12.50	0.176^†^
Baseline CCA-IMT (mm)					
Maximum, left	1.105	±0.382	1.101	±0.410	0.941^†^
Mean, left	0.863	±0.232	0.855	±0.242	0.825^†^
Maximum, right	1.005	±0.348	1.053	±0.405	0.364^†^
Mean, right	0.801	±0.206	0.822	±0.229	0.482^†^

Continuous variables are expressed as mean ± standard deviation (SD) or median (interquartile range), whereas categorical variables are presented as absolute values and percentages

*LAA* large artery atherosclerosis, *SVO* small vessel occlusion, *TIA* transient ischemic attack, *NIHSS* NIH Stroke Scale, *mRS* modified Rankin scale, *WBC* white blood cell, *HDL* high-density lipoprotein, *LDL* low-density lipoprotein, *CCA-IMT* common carotid artery intima-media thickness

* *p* values are for Chi-squared test unless indicated

^†^ *p* value is for Student’s *t* test

^‡^ *p* value is for Fisher’s exact test

^§^ *p* value is for Mann–Whitney test

### Carotid IMT

Maximum and mean IMTs in the left and right CCAs were reduced significantly in the cilostazol group compared to those in the control group. ANCOVA adjusted for age, gender, stroke subtype, location, previous stroke, hypertension, diabetes, hyperlipidemia, current smoking, statin administration, body mass index, glycated hemoglobin, total cholesterol, HDL cholesterol, triglyceride, LDL cholesterol, and interval showed that cilostazol significantly reduced the maximum and mean CCA-IMTs, whereas other antiplatelets did not (Table 2). In addition, there was a difference between the mean changes of the mean CCA-IMTs in low-dose (100 mg/day) cilostazol subjects and those of control and high-dose (200 mg/day) cilostazol subjects, but it was not consistently significant because of small cases (Supplementary Table 2).

**Table 2:** Changes in mean and maximum CCA-IMT values between baseline and follow-up

	Cilostazol (*n* = 101)		Control (*n* = 107)		Cronbach’s alpha	ICC	*p* value
Left maximum CCA-IMT							0.001
Baseline	1.105	±0.382	1.101	±0.410	0.929	0.833	
Follow-up	1.057	±0.396	1.122	±0.447	0.961	0.909	
Mean change	–0.048	±0.186	0.022	±0.163			
Left mean CCA-IMT							<0.001
Baseline	0.863	±0.232	0.855	±0.242	0.934	0.851	
Follow-up	0.810	±0.220	0.879	±0.278	0.958	0.912	
Mean change	–0.052	±0.102	0.023	±0.112			
Right maximum CCA-IMT							0.001
Baseline	1.005	±0.348	1.053	±0.405	0.919	0.815	
Follow-up	0.968	±0.313	1.103	±0.489	0.923	0.840	
Mean change	–0.037	±0.173	0.050	±0.200			
Right mean CCA-IMT							<0.001
Baseline	0.801	±0.206	0.822	±0.229	0.912	0.812	
Follow-up	0.764	±0.183	0.865	±0.287	0.940	0.877	
Mean change	–0.038	±0.106	0.042	±0.139			

Values are mean ± SD. Comparisons of IMTs during treatment with baseline values were performed by ANCOVA adjusted for age, sex, stroke subtype, location, previous stroke, hypertension, diabetes, hyperlipidemia, current smoking, statin, body mass index, glycated hemoglobin, total cholesterol, HDL cholesterol, triglyceride, LDL cholesterol, and interval

*ICC* intraclass correlation coefficient

**Fig. 2:**
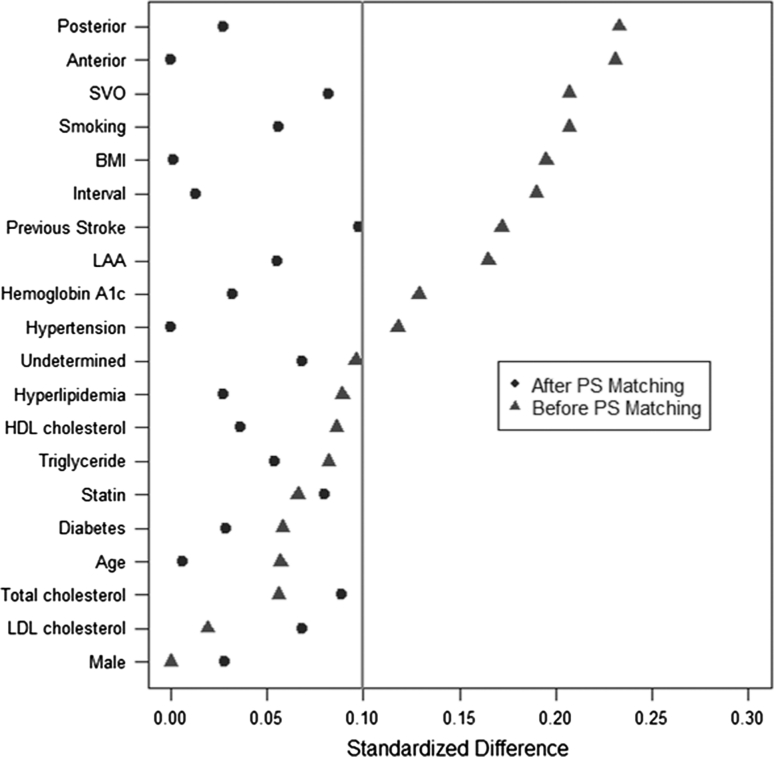
Absolute standardized differences of covariates before and after propensity score matching. *SVO* small vessel occlusion, *BMI* body mass index, *LAA* large artery atherosclerosis

Matched sets by propensity score analyses generated 76 sets of cilostazol and control pairs. Absolute standardized differences were less than 10 % for all covariates, which suggests a successful matching (Fig. 2). After the propensity score matching, the values of both maximum and mean CCA-IMTs were also significantly improved in the cilostazol group in comparison with the control group (Table 3).

**Table 3:** Changes in mean and maximum CCA-IMT values between baseline and follow-up in the matched group

Mean change	Cilostazol (*n* = 76)		Control (*n* = 76)		*p* value
Left maximum CCA-IMT	–0.050	±0.188	0.020	±0.172	0.012
Left mean CCA-IMT	–0.057	±0.094	0.024	±0.109	<0.001
Right maximum CCA-IMT	–0.025	±0.188	0.054	±0.184	0.006
Right mean CCA-IMT	–0.033	±0.104	0.050	±0.130	<0.001

Comparisons of IMTs during treatment with baseline values were performed by paired *t* test

Mean change = follow-up IMT − baseline IMT

### Other parameters

We analyzed some laboratory data at baseline and follow-up. Total cholesterol, LDL cholesterol, and triglyceride were all decreased, while HDL cholesterol were increased in both groups (Table 4). Among the changes of lipid profile between baseline and follow-up, only HDL cholesterol was significantly improved in the two-way ANOVA adjusted for administration of statins (*p* = 0.006).

**Table 4:** Changes in total cholesterol, LDL and HDL cholesterol, and triglycerides between baseline and follow-up

	Cilostazol		Control		*p* value
Total cholesterol (mg/dl)					0.557
Baseline	188.20	±41.34	190.42	±37.59	
Follow-up	169.11	±35.53	167.69	±35.30	
Mean change	–21.53	±43.01	–23.17	±48.95	
HDL cholesterol (mg/dl)					0.006
Baseline	41.46	±11.00	40.51	±11.02	
Follow-up	53.86	±15.00	46.95	±12.95	
Mean change	11.50	±15.59	6.29	±10.82	
Triglyceride (mg/dl)					0.212
Baseline	141.91	±70.43	149.37	±106.87	
Follow-up	113.63	±54.23	141.76	±77.92	
Mean change	–29.85	±65.23	–9.94	±105.99	
LDL cholesterol (mg/dl)					0.838
Baseline	117.40	±35.68	118.08	±33.82	
Follow-up	97.24	±31.40	100.83	±31.83	
Mean change	–21.00	±35.07	–17.19	±42.56	
HbA1c (%)					0.750
Baseline	6.05	±1.23	6.22	±1.37	
Follow-up	6.31	±0.93	6.51	±1.38	
Mean change	0.13	±1.18	0.19	±1.21	

*p* value by two-way ANOVA adjusted for administration of statins

Ischemic stroke recurrence occurred in four (4.0 %) and five (4.7 %) in the cilostazol and control groups, respectively (Table 5).

**Table 5:** Clinical outcomes during the observation period in both groups

	Cilostazol (*n* = 101)	Control (*n* = 107)	*p* value*
Ischemic stroke	4 (4.0 %)	5 (4.7 %)	>0.999
Transient ischemic attack	1 (1.0 %)	2 (1.9 %)	>0.999
Angina or myocardial infarction	6 (5.9 %)	1 (0.9 %)	0.059
Major bleeding^a^	0 (0.0 %)	1 (0.9 %)	>0.999

* *p* values are for Fisher’s exact test

^a^Hemorrhagic stroke and hemorrhage requiring hospital admission and/or transfusion

## Discussion

This is the first case-controlled study investigating the effects of cilostazol for an average of 2.1 years on the progression of carotid IMT in patients with ischemic stroke. Our results reveal that cilostazol may reduce carotid IMT and increase the HDL cholesterol level in ischemic stroke patients.

Ultrasound examination of carotid arteries is a simple and noninvasive method to evaluate early carotid atherosclerosis by measuring IMT or by direct visualization of atherosclerotic plaque. CCA-IMT itself is a more reproducible measure for cardiovascular risk assessment and intervention studies than those at other sites of the carotid artery, so we did not include carotid bifurcation or ICA IMT [32]. It has also been shown that the changes in carotid IMT measurements over time correlate with the occurrence of future cerebrovascular events [33]. However, the subjects in these previous studies were usually healthy patients with diabetes or coronary disease. There is limited data regarding carotid IMT regression trials in ischemic stroke patients [1, 6, 16, 17, 21, 24, 28, 30, 31]. Ischemic stroke patients have a three- to fivefold higher risk of early recurrence if the stroke was caused by small or large artery disease [22]. The study of carotid atherosclerosis in ischemic stroke patients is valuable not only because it has a direct relationship but because it can help in assessing combined systemic atherosclerosis including peripheral artery disease [5, 12].

There have been many pharmaceutical trials using lipid-lowering drugs such as different kinds of statins and niacin, peroxisome proliferator-activator receptors such as rosiglitazone and pioglitazone, anti-hypertensitivity drugs such as ACE inhibitors, calcium channel antagonists, and β-blockers, all of which confirm carotid IMT regression [3, 13, 19, 20, 26, 29, 31]. However, there have been few studies on the effects of antiplatelet agents on the progression of carotid IMT. Aspirin and ticlopidine (a thienopyridine derivative) have been studied to assess their abilities to attenuate carotid IMT progression [17]. Although the antiplatelet actions are mediated by different mechanisms, these antiplatelets showed low efficacy for attenuating the progression of carotid IMT of type 2 diabetes patients. On the other hand, studies evaluating the effects of cilostazol therapy on carotid IMT revealed nearly complete prevention or a decrease in carotid IMT after 1–2 years of cilostazol treatment [1, 16, 24, 28]. The results of these studies indicate that cilostazol is a more potent antiplatelet agent than the others for regression of carotid IMT.

Cilostazol is a different type of antiplatelet agent that inhibits type 3 phosphodiesterase and decreases thromboxane formation by enhancement of the platelet/cAMP level. The increase in cAMP level leads to an inhibition of platelet aggregation, vasodilation, and vascular smooth muscle cell proliferation, an increase in heart rate and contractile force, and an improvement in lipid metabolism [9, 11]. Because large quantities of phosphodiesterase 3 are found in vascular smooth muscle cells, cilostazol can inhibit smooth muscle cell proliferation. Due to these effects, cilostazol has been consistently shown to reduce coronary restenosis in patients with stent implantation and intracranial arterial stenosis in acute stroke patients with symptomatic middle cerebral artery or basilar artery stenosis [8, 18]. Dipyridamole, a phosphodiesterase 5 inhibitor, also has an antiproliferative effect, and extended-release dipyridamole with aspirin (Aggrenox) showed an effect on hemodialysis graft potency [7]. However, Aggrenox is not available in Korea, so we could not directly compare the effects of a phosphodiesterase 3 inhibitor with those of a phosphodiesterase 5 inhibitor in ischemic stroke patients. A few experimental studies have revealed that the inhibition of phosphodiesterase 3 can elicit potent anti-mitogenic and anti-proliferative activities [2, 11].

Although the anti-proliferative mechanism of cilostazol has not yet been established, the beneficial effects on lipoprotein metabolism may play a role in carotid IMT regression. In our study, all lipid profiles and glycated hemoglobin were significantly improved in both groups. This may be caused by the abnormality of baseline laboratory findings in acute stages of ischemic stroke and aggressive treatment of dyslipidemia and diabetes after stroke. Baseline blood tests were performed within 1 week after ischemic stroke, and at the follow-up examination, more than 50 % of all patients were using statins. Nevertheless, the improvement in HDL cholesterol was significantly greater than that in the cilostazol group. Several studies on cilostazol have revealed that the drug can lead to decreases in triglyceride and LDL cholesterol levels and an increase in HDL cholesterol [10, 16, 25]. Previous trials evaluating the use of niacin with statins demonstrated their superiority for IMT regression acquired by an increase in HDL cholesterol rather than a decrease in LDL cholesterol [31]. In our study, no subject received niacin, but an increase in HDL cholesterol by cilostazol yielded an indirect effect on carotid IMT regression. In fact, LDL cholesterol and triglyceride levels may be influenced by cilostazol, but aggressive treatment with statins or fenofibrates to reduce LDL cholesterol or triglyceride level may offset the effect of cilostazol. Because the rates of patients with statin administration were not significantly different, we could not perform further analysis.

Our study does have some limitations. First, our study design was not a randomized controlled trial, and our subjects were recruited from a single center and were thus not representative of the general population. However, demographic findings and initial CCA-IMT values of our subjects were very similar in both groups. Secondary analyses using propensity score matching revealed similar results. In addition, carotid ultrasounds were performed by one sonographer, and the IMT measurements were read by two independent observers blinded to clinical information using semi-automated software. Second, some patients allocated to the cilostazol group have taken other antiplatelets as well as cilostazol. We cannot entirely exclude the possibility that concomitant antiplatelet use in the cilostazol group could influence and weaken the results of our study. However, the prevalence of other antiplatelet use was higher in control group, so that such bias, if any, might not be enough to affect or change our result. Third, the Mannheim consensus recommends that IMT and plaque measurements should be included as secondary endpoints if clinical outcome parameters are defined in the study [32]. However, we did not select clinical outcome as a primary outcome because clinical events including cardiovascular or cerebrovascular attacks and bleeding complication can alter the subject’s antiplatelet regimen and hospital follow-up. To compensate for this weak point, we adopted propensity score matching analysis. Fourth, some patients had plaques in the carotid bifurcation, ICA, and sometimes in the CCA. Further study of the volumetric evaluation of carotid plaque is needed to assess the direct effects of cilostazol on carotid atherosclerosis.

Our results indicate that cilostazol is more beneficial to the regression of carotid IMT in symptomatic ischemic stroke patients than are other antiplatelet agents. In addition, cilostazol can increase serum HDL cholesterol level and can reduce atherosclerotic proliferation. This suggests that cilostazol is a powerful therapeutic agent for controlling carotid atherosclerosis in ischemic stroke patients.

## Electronic supplementary material

Below is the link to the electronic supplementary material.

Supplementary material 1 (PDF 1759 kb)
