# Supplementary material for: Effects of cilostazol against the progression of carotid IMT in symptomatic ischemic stroke patients
Source: J Neurol. 2012 Jul 21;260(1):122–30. doi: 10.1007/s00415-012-6599-y (PMC3535378; doi:10.1007/s00415-012-6599-y)

Supplementary Table 1 Antiplatelet treatment regimen in cilostazol and control groups

	Cilostazol (N=101)	Control (n=107)	P value*
Cilostazol only	14 (13.9%)	0 (0.0%)	<0.001
Aspirin	35 (34.7%)	22 (20.6%)	0.023
Triflusal	18 (17.8%)	13 (12.1%)	0.251
Clopidogrel	27 (26.7%)	31 (29.0%)	0.719
Aspirin + Clopidogrel	6 (5.9%)	20 (18.7%)	0.005
Triflusal + Clopidogrel	1 (1.0%)	3 (2.8%)	0.622†
Aspirin + Triflusal	0 (0.0%)	18 (16.8%)	<0.001

* P-values are for Chi-squared test unless indicated.

† P-value is for Fisher's exact test.

Supplementary Table 2 Changes in mean and maximum CCA-IMT values between baseline and follow-up

	Cilostazol 100mg/d (n=29)		Control (n=107)		P-value†	Cilostazol 200mg/d (n=72)		P-value†
Left maximum CCA-IMT								
Baseline	1.065	±0.373	1.101	±0.410		1.121	±0.386	
Follow-up	1.049	±0.434	1.122	±0.447		1.060	±0.382	
Mean change	–0.016	±0.174	0.022	±0.163	0.440	–0.061	±0.190	0.001
Left mean CCA-IMT								
Baseline	0.842	±0.238	0.855	±0.242		0.871	±0.230	
Follow-up	0.803	±0.211	0.879	±0.278		0.813	±0.225	
Mean change	–0.038	±0.065	0.023	±0.112	0.013	–0.058	±0.113	<0.001
Right maximum CCA-IMT								
Baseline	0.974	±0.383	1.053	±0.405		1.018	±0.336	
Follow-up	0.989	±0.344	1.103	±0.489		0.960	±0.302	
Mean change	0.015	±0.154	0.050	±0.200	0.365	–0.057	±0.177	0.004
Right mean CCA-IMT								
Baseline	0.778	±0.199	0.822	±0.229		0.810	±0.209	
Follow-up	0.784	±0.195	0.865	±0.287		0.755	±0.179	
Mean change	0.006	±0.081	0.042	±0.139	0.171	–0.055	±0.110	<0.001

† P-value by Dunnett-Hsu's Multiple Comparisons of ANCOVA

Values are mean±SD. Comparisons of IMTs during treatment with baseline values were performed by ANCOVA adjusted for age, sex, stroke subtype, location, previous stroke, hypertension, diabetes, hyperlipidemia, current smoking, statin, body mass index, glycated hemoglobin, total cholesterol, HDL cholesterol, triglyceride, LDL cholesterol, and interval.

Supplementary Fig 1 The image capturing procedure of carotid IMT at baseline (a) and follow-up (b) and IMT measurement by using the semi-automated software, Intima Scope (c).

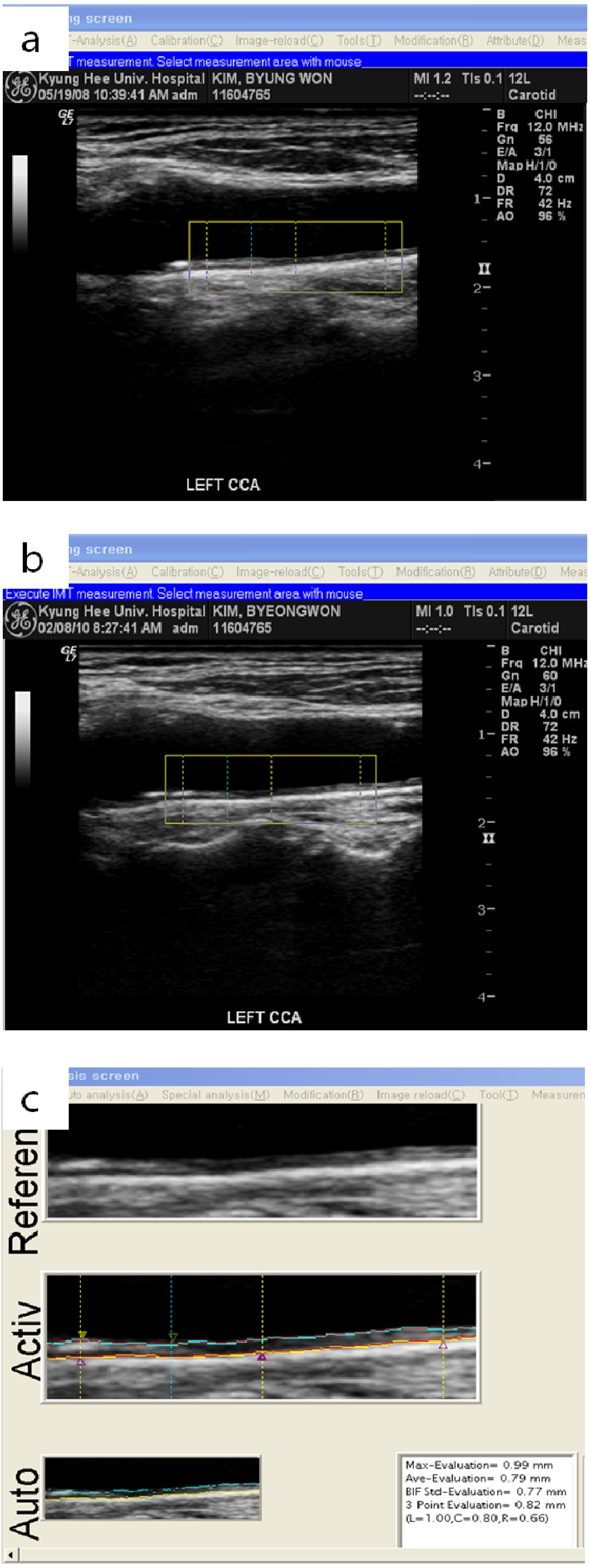

Supplement: Supplementary file 1 — Supplementary material 1 (PDF 1759 kb) [file 415_2012_6599_MOESM1_ESM.pdf]
